# The Antioxidant Guaiacol Exerts Fungicidal Activity Against Fungal Growth and Deoxynivalenol Production in *Fusarium graminearum*

**DOI:** 10.3389/fmicb.2021.762844

**Published:** 2021-11-15

**Authors:** Tao Gao, Yao Zhang, Jianrong Shi, Sherif Ramzy Mohamed, Jianhong Xu, Xin Liu

**Affiliations:** ^1^Jiangsu Key Laboratory for Food Quality and Safety-State Key Laboratory Cultivation Base, Ministry of Science and Technology, Institute of Food Safety and Nutrition, Jiangsu Academy of Agricultural Sciences, Nanjing, China; ^2^Key Laboratory for Control Technology and Standard for Agro-Product Safety and Quality, Ministry of Agriculture and Rural Affairs, Institute of Food Safety and Nutrition, Jiangsu Academy of Agricultural Sciences, Nanjing, China; ^3^Key Laboratory for Agro-Product Safety Risk Evaluation (Nanjing), Ministry of Agriculture and Rural Affairs, Institute of Food Safety and Nutrition, Jiangsu Academy of Agricultural Sciences, Nanjing, China; ^4^Collaborative Innovation Center for Modern Grain Circulation and Safety, Institute of Food Safety and Nutrition, Jiangsu Academy of Agricultural Sciences, Nanjing, China; ^5^School of Food Science And Engineering, Jiangsu Ocean University, Lianyungang, China; ^6^School of Food and Biological Engineering, Jiangsu University, Zhenjiang, China; ^7^Department of Food Toxicology and Contaminant, National Research Centre of Egypt, Giza, Egypt

**Keywords:** *Fusarium graminearum*, guaiacol, intracellular Ca^2+^, deoxynivalenol, antioxidant

## Abstract

The main component of creosote obtained from dry wood distillation—guaiacol—is a natural antioxidant that has been widely used in pharmaceutical and food preservation applications. However, the antifungal mechanism of guaiacol against phytopathogens remains unclear. In this study, we found that guaiacol exerts inhibitory effects against mycelial growth, conidial formation and germination, and deoxynivalenol (DON) biosynthesis in *Fusarium graminearum* in a dose-dependent manner. The median effective concentration (EC_50_) value of guaiacol for the standard *F. graminearum* strain PH-1 was 1.838 mM. Guaiacol strongly inhibited conidial production and germination. The antifungal effects of guaiacol may be attributed to its capability to cause damage to the cell membrane by disrupting Ca^2+^ transport channels. In addition, the decreased malondialdehyde (MDA) levels and catalase (CAT), peroxidase (POD), and superoxide dismutase (SOD) activity by guaiacol treatment indicate that guaiacol displays activity against DON production by modulating the oxidative response in *F. graminearum.* Taken together, this study revealed the potentials of antioxidant in inhibiting mycotoxins in *F. graminearum*.

## Introduction

Wheat is the most important food crop in the world and ranks first in terms of planting area and yield. More than one-third of the world’s population consumes wheat products as their staple food (Giraldo et al., 2019). *Fusarium* head blight (FHB), an important disease at the spike stage caused by the *Fusarium graminearum* species complex (FGSC) that affects the yield and quality of wheat (Dean et al., 2012), mainly occurs in wet and rainy areas (Starkey et al., 2007). In China, FHB is an economically important disease that has spread over a broad area to wheat-growing areas in the middle and lower reaches of the Yangtze River and spring wheat areas in the northeast of China (Qiu et al., 2014). FHB not only seriously affects the wheat yield, but is also related to the accumulation of toxic secondary metabolites, such as deoxynivalenol (DON) and zearalenone (ZEN), during disease infestation; these metabolites remain and accumulate in the seeds, seriously affecting the quality and safety of grains and endangering human and animal health (Pinton and Oswald, 2014). Due to the widespread presence of the toxin, many countries, including China, have established maximum levels of DON allowed in wheat and other grain products, and the control of FHB and DON pollution is urgently needed.

Currently, chemical fungicides such as the sterol demethylation inhibitors tebuconazole and prothioconazole (Chen et al., 2019), the novel myosin inhibitor phenamacril and the carboxamide fungicide pydiflumetofen (Sun et al., 2019) are mainly used in the production of wheat to control FHB (Sevastos et al., 2013). However, *F. graminearum* populations can easily develop resistance to these chemical fungicides and chemical fungicide residues with high toxicity pose a potential risk to the environment and human health. It was reported that the DON content in diseased wheat grains is also significantly increased in areas with severe occurrence of carbendazim resistance (Adedeji et al., 2017), and methoxyacrylate fungicides also stimulate *Fusarium* to produce more of the toxin DON (Duan et al., 2020). Therefore, exploring new, efficient and low-risk agents that can not only control disease but also inhibit toxin biosynthesis has become a key breakthrough in the effective control of FHB.

Guaiacol (2-methoxyphenol), also called methylcatechol, is a white or yellow crystalline material or transparent yellow oily liquid that is mainly found in guaiac resin or pine oil in nature; in addition, guaiacol is the main component of creosote, which is obtained from dry wood distillation (Mohan et al., 2006). Guaiacol is used in a wide range of industrial applications and often used to produce a variety of fragrances, such as eugenol, vanillin and artificial musk (Hiscox and Boddy, 2017). Guaiacol is also widely used in medicine for the synthesis of guaiacol benzoate (potassium guaiacol sulfonate), as a local anesthetic or antiseptic, and in the preparation of oral solutions for expectoration and the treatment of indigestion (De Souza Araújo et al., 2018). However, few studies on the use of guaiacol to inhibit plant pathogens have been reported (Yang et al., 2013). Some essential oils containing substituted phenols, such as eugenol, thymol, carvacrol and guaiacol, have strong antimicrobial and antioxidant activities (Atanasova-Penichon et al., 2016; Zhao et al., 2018). Vanilla extract and vanillin, which are rich in guaiacol, can inhibit the growth of *Alternaria alternata* (Romero-Cortes et al., 2019). Some plant acids or wood vinegar solutions with antibacterial or antifungal effects are also rich in guaiacol (De Souza Araújo et al., 2018). A recent study suggested that wheat straw vinegar can significantly inhibit the growth of *F. graminearum* and DON production, which the active ingredient is guaiacol compounds (Gao et al., 2020), but the antimicrobial activity of guaiacol against *F. graminearum* is poorly understood.

Therefore, in this work, we identified the inhibitory activity of guaiacol against the growth of *F. graminearum* and its production of DON. Guaiacol-induced disruption of the cell membrane in *F. graminearum* was studied by physiological assessment and microscopic observation. All of these results can improve our understanding of the action mode of guaiacol, which will assist us in providing new reference data for the management of FHB caused by *F. graminearum*.

## Materials and Methods

### Media, Strains, Chemicals, and Culture Conditions

The standard strain of *F. graminearum* was PH-1 (NRRL31084), which is used to investigate the detailed physiological and biochemical responses under guaiacol treatment (Gu et al., 2015). The strains were grown on Potato Dextrose Agar (PDA) medium and Carboxymethyl Cellulose (CMC) medium for mycelial growth assay and sporulation assay, respectively. Trichothecene biosynthesis inducing (TBI) media was used for the DON production assay (Gardiner et al., 2009). Yeast extract peptone dextrose (YEPD) medium was used for conidial germination assays and fungal culture prior to quantitative real-time RT-PCR (qRT-PCR) analysis for gene expression (Gao et al., 2016a). Guaiacol (a.i. 99%;100 ml; Shanghai Macklin Biochemical Co., Ltd.) was stored at 4°C in darkness.

### Determination of the *in vitro* Activity of Guaiacol Against *Fusarium graminearum*

The mycelial growth of PH-1 in PDA with or without guaiacol was measured after incubation for 3 days at 25°C. Growth inhibition as a percent of the control was calculated. The median effective concentration (EC_50_) of guaiacol was calculated based on linear regression of colony diameter vs. log-transformed fungicide concentration (Thomulka et al., 1996). The bioassay data were obtained as the mean of three replicates. The morphology of mycelia treated with guaiacol was observed by scanning electron microscopy (SEM, EVO-LS10, ZEISS, Jena, Germany), and sample drying was carried out according to a previously described method (Gao et al., 2016b).

For the conidiation assay, five 5-mM mycelial plugs were taken from the edge of a 3-day-old colony into 150-ml flasks containing 50 ml of CMC culture. The flasks were incubated at 25°C for 5 days with shaking (175 rpm). The number and morphology of the conidia were determined with a hemocytometer and microscope, respectively (Duran et al., 2010). Conidia (10^5^) of *F. graminearum* strain PH-1 were inoculated into 30 ml of YEPD medium with or without guaiacol. Microscopic examination was carried out using germ tubes germinated from spores for 2, 4, 6, 8, 10, 12, and 24 h.

### Determination of Relative Conductivity and the Glycerol Content

The relative conductivity and glycerol content of guaiacol-treated *F. graminearum* were measured according to a method described by Gao et al. (2016b). Conidia (10^5^) of PH-1 were placed in 250-ml flasks containing 100 ml of YFPD medium at 25°C with shaking (175 rpm). After 24 h, some of the flasks were treated with guaiacol and shaken for an additional 24 h. Then, the mycelia were washed and suspended at 0.5 g of mycelia per sample in 20 ml of double-distilled water. A conductivity meter (CON510 Eutech/Oakton, Singapore) was used to measure the electrical conductivity after 0, 5, 10, 20, 40, 60, 80, 100, 120, 140, 160, and 180 min. After 180 min, the final conductivity of the mycelia was measured after boiling for 5 min. The relative conductivity was calculated with the following formula:


Relativeconductivity(%)=Conductivity/Finalconductivity×100

Glycerol in the mycelia was measured with a glycerol detection kit (Beijing Applygen Technologies Inc., Beijing, China), and mycelia were treated as described above.

### Measurement of Antioxidant Enzyme Activity and Lipid Oxidation Levels

To observe the impact of guaiacol on the antioxidant system in *F. graminearum*, peroxidase (POD), superoxide dismutase (SOD), catalase (CAT), and malondialdehyde (MDA) levels were compared. Conidia (10^5^) of PH-1 were cultured on YEPD medium for 24 h and then treated with guaiacol at different concentrations for another 24 h. A peroxidase assay kit (A084-3-1), catalase assay kit (A007-1-1) and superoxide dismutase assay kit (A001-1-1) from Nanjing Jiancheng Bioengineering Institute were used to measure enzyme activities. An MDA detection kit (D799761; Shanghai Sangon Biotech Institute, Shanghai, China) was used to determine the MDA level based on the spectrophotometric determination of the reaction between MDA and 1,3-diethyl-2-thiobarbituric acid (TBA) assisted by trichloroacetic acid (TCA) (Yang et al., 2012). Enzymatic activities and the MDA content were normalized according to their mycelial dry weight.

### Determination of Intracellular Ca^2+^ Balance

The mycelia were treated with guaiacol as described above, and the calcium indicator Fluo-3-acetoxymethyl (Fluo-3-AM; S1056; Shanghai Beyotime Biotechnology Institute, Shanghai, China) was used for detecting the changes of Ca^2+^ concentration in the mycelia with guaiacol-treated. All mycelia were loaded with 5 μM Fluo-3-AM ester, a membrane-permeable calcium indicator, for 40 min at 37°C. After loading with the Fluo-3 dye, the mycelia were washed with ddH_2_O. Fluorescence was observed using a PerkinElmer UltraVIEW VoX system equipped with a 60 × objective. Fluo-3 was excited by argon laser light at 488 nm (Li et al., 2009).

### Real Time Quantitative Reverse Transcription

The real-time quantitative reverse transcription-polymerase chain reaction (qRT-PCR) was selected to quantify the expression levels of Ca^2+^-ATPase (PMCA) genes. The sequences of four tested genes (accession number: FGSG_00508, FGSG_04245, FGSG_04919, and FGSG_08985) were retrieved from NCBI Database^1^ for the design of primers. Total RNA was extracted from the mycelia of each sample by using TRIzol (Invitrogen, Waltham, MA, United States) according to the manufacturer’s instructions (Gao et al., 2016a). The first-strand cDNA synthesized with the PrimeScript^®^ RT reagent kit (TaKaRa, Kusatsu, Japan) was used as template for qRT-PCR analysis (Roche Molecular Systems, LightCycler^®^ 96, Life Technologies, United States). The primers used for amplifying the target genes are listed in Supplementary Table 1. To standardize the results, the relative abundance of elongation factor 1-α (EF1-α, FGSG_08811) was also determined and used as the internal standard.

### Quantification of Deoxynivalenol Production and *Tri5* and *Tri6* Expression Levels

To quantify DON production *in vitro*, conidia (10^5^) of PH-1 were inoculated in 30 ml of liquid trichothecene biosynthesis-inducing (TBI) medium with or without guaiacol at different concentrations and cultured at 28°C for 7 days in the dark. Then, the solution was filtered, and the mycelia were dried and weighed. The filtrate was redissolved in methanol for DON analysis, and the mycelia were used for RNA extraction. The expression levels of two trichothecene biosynthesis genes (Tri5 and Tri6) were determined with real-time quantitative reverse transcription-polymerase chain reaction (qRT-PCR). The primers used to amplify the *Tri5* and *Tri6* genes are listed in Supplementary Table 1. The amount of DON in each sample was determined using a high-performance liquid chromatography-mass spectrometer/mass spectrometer (HPLC-MS/MS) system (a Shimadzu 30A LC system coupled to a Triple Quad 6500+, Sciex, United States). The mass spectrometric parameters were set according to a previously described method (Dong et al., 2016). The experiment was repeated two times.

To quantify DON production in wheat, 20 g of finely ground samples were extracted with 80 ml of acetonitrile:water (84:16, v/v) for 60 min at 200 rpm on an orbital shaker. The extracts were filtered and passed through a MycoSep 226 AflaZon + multifunctional column (Romer Labs, Beijing, China), and 4 ml of the purified extract was evaporated to dryness under a nitrogen stream. The residue was dissolved in 1 ml of methanol:water (40:60, v/v), followed by centrifugation at 10,000 rpm and subsequent analysis by LC-MS/MS analysis of DON (Dong et al., 2016). The recovery of DON in wheat ranged from 82.1 to 95.7%. The limit of detectionm (LOD) quantification (LOQ) of DON were estimated based on signal-to-noise ratios of 3/1 (10 μg/kg) and 10/1 (30 μg/kg), respectively.

### Field Trials

Field trials were conducted in the village of Jinci in Zhuzhen (Luhe District, Nanjing, Jiangsu Province, China). The local common wheat cultivar, Zhengmai 10, which is moderately resistant to *F. graminearum*, was chosen to test the efficacy of guaiacol. The three following treatments were applied: for treatments 1 and 2, guaiacol diluted 1:200 (40 mM Gua) and 1:100 (80 mM Gua), respectively, was sprayed during the flowering period, and for treatment 3, water was sprayed as a control. Each treatment was performed in four replications, and the plot area was 5 m × 5 m. The experiments were conducted as a randomized complete block design with a 1-m protection row surrounding each plot.

After 7 days, all treatments were applied for a second time. It should be noted that all the treatments were conducted on a sunny day in the late afternoon to avoid the spray being washed off of the plants by rainfall. The experimental site was naturally infected. Two weeks after the final treatments, wheat heads were collected from five sampling locations in each plot, and 100 heads of wheat were collected at each sampling location. Finally, the disease incidence (percentage of diseased heads), disease index (DI), and control efficacy were calculated. The DI in the wheat heads was assessed using five evaluation classes according to the percentage of the wheat head surface that showed the symptoms of FHB (0: 0%; 1: 1–25%; 2: 26–50%; 3: 51–75%; and 4: > 75%). The DI of each plot was calculated using the following formula: [(number of wheat heads in each class × class number)/(total number of wheat heads × 4)] × 100.

### Statistical Analysis

All data are expressed as the mean ± standard deviation (SD). The statistical significance of differences was determined by the unpaired Student’s *t*-test with GraphPad Prism software (La Jolla, CA, United States). A *p*-value less than 0.05 was used to indicate a statistically significant difference.

## Results

### Effect of Guaiacol on the Growth, Conidia Production and Germination of *Fusarium graminearum*

**FIGURE 1:**
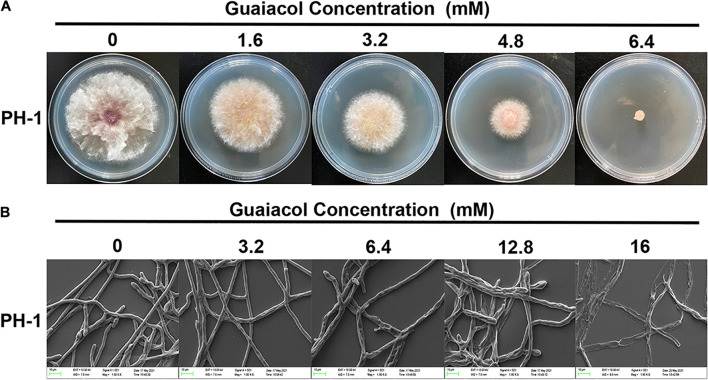
Guaiacol inhibits the mycelial growth of *F. graminearum*. **(A)** Strain PH-1 was cultivated on PDA plates containing guaiacol (0–6.4 mM) for 3 days for photographing and the measurement of the diameter of colonies; **(B)** SEM observation of the hyphal morphology of *F. graminearum* (PH-1) under guaiacol (0–16 mM) treatment.

Treatment of *F. graminearum* PH-1 with guaiacol at different concentrations showed that guaiacol inhibited hyphal growth in a dose-dependent manner and that 6.4 mM guaiacol completely inhibited mycelial growth (Figure 1A). The EC_50_ value of guaiacol for PH-1 was 1.838 mM. Furthermore, assessment of the mycelial growth of *F. graminearum* by SEM clearly revealed morphological alterations in the hyphae upon guaiacol treatment. Unlike hyphae in the control group, which were regular and smooth, treatment with 16 mM guaiacol led to significant collapse of the hyphae (Figure 1B).

**FIGURE 2:**
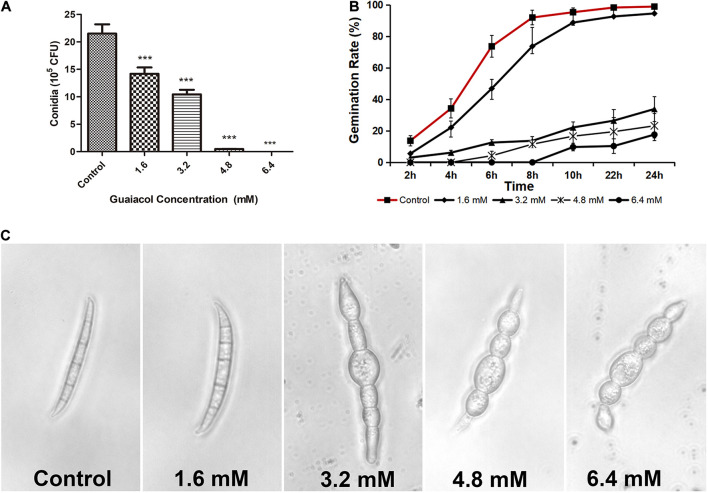
Guaiacol treatment altered conidial morphology, formation and germination. **(A)** Conidia were induced in liquid CMC containing guaiacol (0–6.4 mM) for 4 days, in a shaker at 180 rpm, 25°C with lights on. Then the conidia were counted under microscopy. **(B)** Conidial germination were compared between control and guaiacol treatment at the concentration indicated in the figures. Each treatment has three replicates, ***p < 0.001. **(C)** The conidia were treated with guaiaol at 0, 1.6, 3.2, 4.8, and 6.4 mM for 24 h, respectively. Then the samples were visualized and photographed under light microscope.

The conidial production and germination of *F. graminearum* were also depressed by guaiacol. Compared to that in the control, the number of conidia was significantly decreased by 97.8% upon treatment with 4.8 mM guaiacol (Figure 2A), and guaiacol at 1.6–6.4 mM delayed the process of conidial germination. In the control group, all the conidia germinated completely after 8 h, and the germination rate decreased gradually with increasing guaiacol concentration (Figure 2B). Light microscopy was used to observe the morphology of conidia under guaiacol treatment. After treatment for 24 h, the conidia treated with guaiacol became wider and shorter than those of the control (Figure 2C). All these results suggest that guaiacol had a remarkable effect on hyphae growth and conidial development in *F. graminearum*.

### Guaiacol Induced Cell Membrane Injury of *Fusarium graminearum*

**FIGURE 3:**
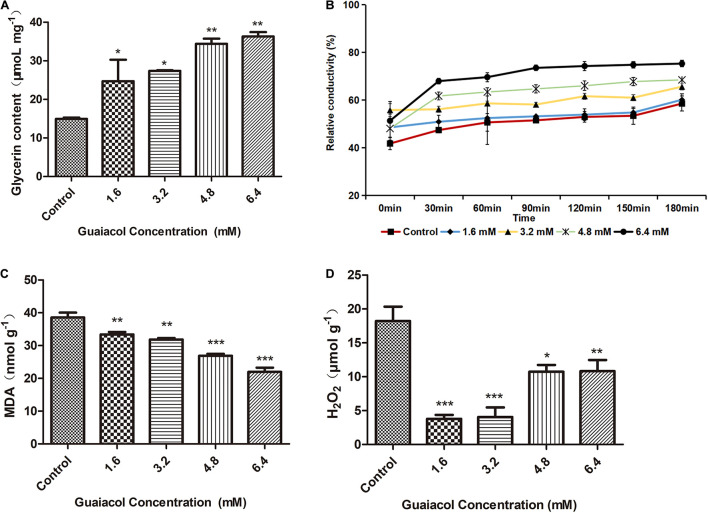
Effect of guaiacol on **(A)** glycerin content, **(B)** the relative conductivity, **(C)** MDA content, and **(D)** H_2_O_2_ content of the mycelia of *F. graminearum* (PH-1). Mycelia treated with guaiacol at 0, 1.6, 3.2, 4.8, and 6.4 mM for 24 h were harvested for the measurements. Each treatment has three replicates, *p < 0.05, **p < 0.01, and ***p < 0.001.

To test the effect of guaiacol on the *F. graminearum* cell membrane, we measured relative conductivity, the MDA concentration, and the glycerol and hydrogen peroxide (H_2_O_2_) content of *F. graminearum* upon guaiacol treatment. Intracellular glycerol has been shown to play an important role in the response of fungi to osmotic stress (Duran et al., 2010). In the present study, we found that the glycerol content was significantly higher in guaiacol-treated *F. graminearum* compared to the control group (Figure 3A), suggesting that glycerol accumulation was induced by guaiacol to maintain osmotic balance. Treatment with guaiacol resulted in a remarkable increase in the relative conductivity of *F. graminearum* compared to that of the control group (Figure 3B), suggesting that guaiacol can induce electrolyte leakage by enhancing cell membrane permeability in *F. graminearum*. Electrolyte leakage resulting from damage to the cell membrane can trigger osmotic stress responses in fungi. The MDA concentration has been frequently used as an index of lipid peroxidation, which indicates oxidative injury of the cell plasma membrane (Tsikas, 2017). In this study, we found that the MDA and H_2_O_2_ content was significantly lower in guaiacol-treated *F. graminearum* compared to the control group (Figures 3C,D), suggesting that the guaiacol-induced cell membrane damage was not related to oxidative damage. Indeed, as a strong oxidant, guaiacol can also effectively remove reactive oxygen species from *F. graminearum.*

### Effect of Guaiacol on the Antioxidant Enzymatic Activities

**FIGURE 4:**
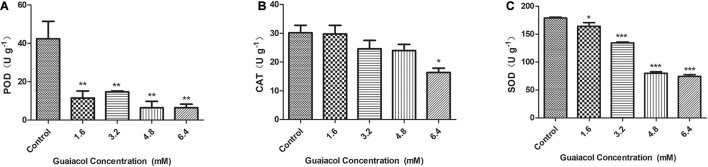
Effects of guaiacol on the antioxidant enzymes activities of POD **(A)**, CAT **(B),** and SOD **(C)** in *F. graminearum*. Mycelia treated with guaiacol at 0, 1.6, 3.2, 4.8, and 6.4 mM for 24 h were harvested for the measurements. Each treatment has three replicates, *p < 0.05, **p < 0.01, and ***p < 0.001.

To gain more information on the possible link between guaiacol and oxidation, POD, CAT and SOD activities were measured in untreated *F. graminearum* samples and *F. graminearum* samples treated with 1.6–6.4 mM guaiacol, followed by 24 h of cultivation. The results shown in Figure 4 demonstrate that the POD, CAT, and SOD activities were also inhibited in the guaiacol-treated samples compared with the untreated samples. These results indicate that guaiacol may restore homeostasis during oxidative stress in *F. graminearum*.

### Effect of Guaiacol on Deoxynivalenol Biosynthesis in *Fusarium graminearum*

**FIGURE 5:**
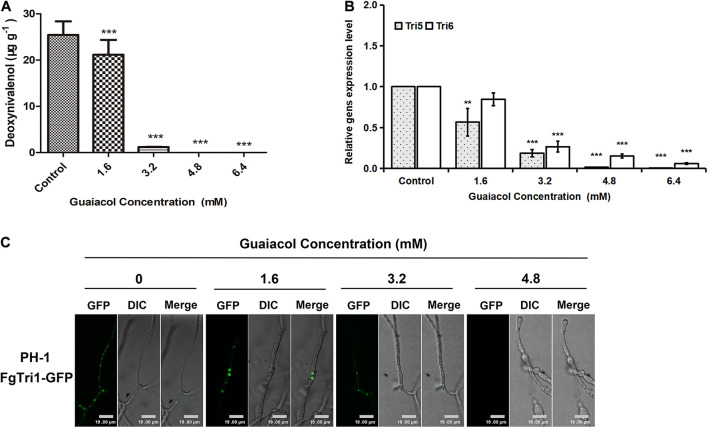
Guaiacol treatment affects DON biosynthesis. **(A)** Equal amounts of conidia were inoculated into liquid TBI containing guaiacol (0, 1.6, 3.2, 4.8, and 6.4 mM) for 7 days at 28°C with lights on in dark. DON production was normalized with the dry mycelium collected from the culture. **(B)** Relative expression of *Tri* genes (*Tri5* and *Tri6*) involved in DON biosynthesis was determined by qRT-PCR. **(C)** DON-toxisome was examined in the hyphae harvested from 3 day-old liquid TBI culture with guaiacol (0, 1.6, 3.2, and 4.8 mM^)^ treatment under fluorescence confocal microscope. Each treatment has three replicates, **p < 0.01, and ***p < 0.001. Bar = 19 μm.

Consistent with the results of morphological analysis, treatment of *F. graminearum* PH-1 spores with guaiacol at different concentrations (0, 1.6, 3.2, 4.8, and 6.4 mM) showed that guaiacol inhibited DON synthesis in a dose-dependent manner (Figure 5A). Furthermore, the expression levels of two trichothecene biosynthesis genes, *Tri5* and *Tri6*, were investigated by qRT-PCR. Compared to that in the untreated control, the relative expression of these two genes was significantly downregulated (*p* < 0.001) in the guaiacol-treated mycelia (Figure 5B).

In *F. graminearum*, *Tri1* encodes the oxygenase cytochrome P-450, which is involved in the late steps of biosynthesis of the trichothecene DON (Zhang et al., 2015). Upon culture under conditions that induce DON biosynthesis (liquid TBI medium), colocalized fluorescent signals were detected in a mutant tagged with Tri1-GFP (ΔTri1:FgTri1-GFP), indicating DON toxisomes; however, 3.2 or 4.8 mM guaiacol treatment strongly inhibited DON toxisome formation, as only faint fluorescent signals were observed in the guaiacol-treated mycelia (Figure 5C).

### Effect of Guaiacol on the Intracellular Ca^2+^ Concentration

**FIGURE 6:**
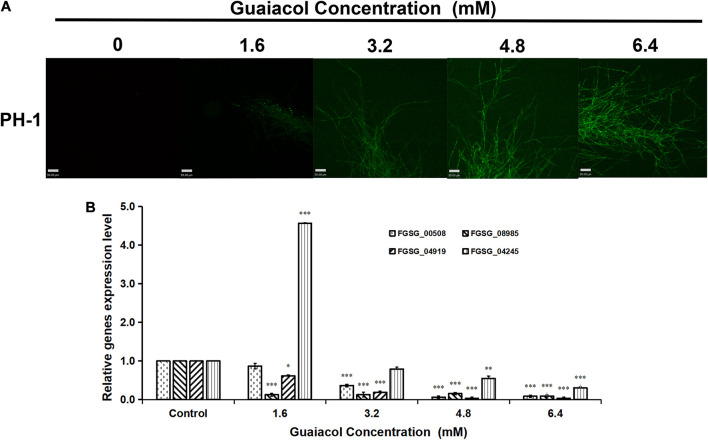
Guaiacol treatment affects intracellular Ca^2+^ concentration. **(A)** Hyphae treated with guaiacol at 0, 1.6, 3.2, 4.8, and 6.4 mM for 24 h were incubated with Fluo-3 AM Calcium indicator. Then, the photos were captured under fluorescence confocal microscope. **(B)** Relative expression of four Ca^2+^-ATPase (PMCA) genes involved in involved in Ca^2+^ transport was determined by qRT-PCR. Each treatment has three replicates, ^∗^*p* < 0.05, ^∗∗^*p* < 0.01, and ^∗∗∗^*p* < 0.001. Bar = 59 μm.

Zhi et al. (2020). found that guaiacol can suppress osteoclastogenesis by inducing Ca^2+^ oscillation. Therefore, in this study, we analyzed changes in the Ca^2+^ concentration in the mycelia of *F. graminearum* treated with guaiacol at different concentrations (0, 1.6, 3.2, 4.8, and 6.4 mM). The Fluo-3-AM-stained hyphae of *F. graminearum* showed more extensive green fluorescence in the presence of guaiacol than in its absence (Figure 6A), indicating that guaiacol treatment led to intracellular Ca^2+^ oscillation. Furthermore, the expression levels of four Ca^2+^-ATPase (PMCA) genes involved in Ca^2+^ transport were determined by qRT-PCR (Bruce, 2018). Compared to that in the untreated control, the relative expression of FGSG_00508, FGSG_04919, FGSG_08985, and FGSG_04245 was significantly downregulated (p < 0.001) in mycelia treated with 6.4 mM guaiacol, although the expression of FGSG_04245 was upregulated upon treatment with guaiacol at low concentrations (Figure 6B).

### Effect of Guaiacol on *Fusarium* Head Blight Control in the Field

**FIGURE 7:**
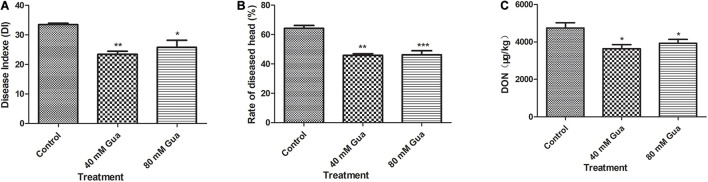
Effects of guaiacol on **(A)** Diseade Indexe, **(B)** Rate of diseased head and **(C)** DON content in the wheat grain. *p < 0.05, **p < 0.01, and ***p < 0.001.

A field experiment was conducted to test the efficacy of guaiacol in controlling FHB and wheat DON contamination. This study was conducted in the dry wheat season; therefore, the FHB and DON concentrations in the grain were relatively low. The results in Figure 7 indicate that guaiacol treatment significantly inhibited FHB infection, and significant decreases in DON content of 17.2 and 23.4% were observed following the Gua-200 and Gua-100 treatments, respectively.

## Discussion

Plant extracts have always been of great interest to researchers because of their great potential to inhibit pathogens as well as their medicinal properties (Ghuman et al., 2019). Recently, some plant extracts with antioxidant properties have also been proposed to inhibit the growth of phytopathogens and mycotoxin production (Ponzilacqua et al., 2019). Studies have shown that antioxidants can significantly inhibit fungal growth and toxin production in the aflatoxin-producing species *Aspergillus flavus* and the fumonisin-producing species *F. verticillioides* and *F. proliferatum* (Ponzilacqua et al., 2019). Due to the lack of resistant wheat varieties and the risk of resistance to commonly used fungicides, the need to develop and apply new green prevention and control technology is urgent. Effective and substantial control of FHB and toxin contamination caused by members of the FGSC remains a major challenge in the global wheat industry (Rampersad, 2020). Here, we propose that the antioxidant guaiacol is effective in suppressing the growth of the important wheat fungus *F. graminearum* in a dose-dependent manner. There have been few reports of guaiacol as a candidate drug for the treatment of some diseases, and its antimicrobial mechanism is not well understood (Kakhlon et al., 2018). Furthermore, the mechanism of its antioxidant activity is also poorly understood. Further elucidation of the mechanism by which guaiacol inhibits fungal growth and DON synthesis is necessary for the efficient application of this compound in disease and toxin control in agriculture.

In this study, guaiacol at very low concentrations impeded the mycelial growth, conidium production and germination of *F. graminearum*. Treatment with 6.4 mM guaiacol (Figure 1) achieved a 100% mortality rate in *F. graminearum.* The antibacterial activity of natural antioxidants has been reported in many papers. Tannic acid and extracts from Chinese galls were reported to inhibit the germination of conidia in local *F. graminearum* isolates from Switzerland (Forrer et al., 2014). In the present study, guaiacol induced swelling and deformation of the conidia, suggesting that guaiacol triggers an osmotic stress response in *F. graminearum*. This phenomenon was verified by the accumulation of glycerol and increase in relative conductivity, which are typical indicators of osmotic stress in fungi. The results of SEM also showed that guaiacol caused the extravasation of intracellular material from mycelia. All these indexes indicate that guaiacol inhibits mycelial growth by destroying the integrity of the *F. graminearum* cell membrane, and a decrease in the MDA content indicated that this destructive effect is independent of lipid peroxidation.

Widespread FHB not only results in substantial economic losses but also contaminates wheat grain with mycotoxins. DON is one of the most prevalent type B trichothecenes and has been indicated to be toxic to eukaryotic cells by inhibiting protein synthesis (Acharyya et al., 2015). Boutigny et al. (2009). reported that ferulic acid, the most abundant phenolic acid in wheat brain, can regulate the accumulation of DON by inhibiting the expression of *Tri* genes. The biosynthesis of DON is regulated by many exogenous environmental factors, and reactive oxygen species (ROS) burst in the plant defense mechanism is also an important factor for the induction of DON biosynthesis. It has been reported that H_2_O_2_ stimulates *F. graminearum* to produce more DON by inducing the expression of multiple *Tri* genes (Ponts, 2015). Therefore, the use of antioxidants can block the response of *F. graminearum* to oxidative stress and reduce the biosynthesis of DON, providing a new strategy for the control of DON pollution. In the present study, DON production was reduced in liquid medium that induced DON biosynthesis, and the expression of DON biosynthetic *Tri* genes was downregulated upon guaiacol treatment. Guaiacol also inhibited the formation of cellular DON toxisomes in *F. graminearum.* Furthermore, the enzymatic activities of CAT, SOD and POD were decreased by guaiacol treatment in *F. graminearum*. All those results indicated that guaiacol displays antifungal activity against DON production by modulating the oxidative response in fungi.

The level of Ca^2+^, a secondary messenger, in fungal cells affects fungal growth, organelle localization and distribution of the cytoskeleton (Harris et al., 2009). The unexpected flow of intracellular Ca^2+^ leads to disorder of the organelle environment and dysfunctional protein synthesis in the endoplasmic reticulum, which can lead to cell apoptosis (Shanahan et al., 2011). Here, the increased green fluorescence signal of Fluo-3-AM-stained hyphae with guaiacol treatment showed that the antifungal mechanism of guaiacol is related to the disturbance of intracellular Ca^2+^. The main elements that affect calcium ion transport in cells are Ca^2+^-ATPase (PMCA) and the Na^+^/Ca^2+^ ion exchanger (NCX) on the membrane (Brini and Carafoli, 2011). NCX is common in animal cells but has not been reported in fungal genomes. PMCA has a high affinity for Ca^2+^ and can excrete excess Ca^2+^ out of the cell or transport it to the endoplasmic reticulum and Golgi apparatus to maintain a low Ca^2+^ concentration in the cell (Brini et al., 2017). In the present study, the decreased expression of four PMCA genes induced by guaiacol also suggests that guaiacol can disrupt the Ca^2+^ transport pathway.

Collectively, these findings demonstrate that the natural antioxidant guaiacol displayed inhibitory effects against mycelial growth, conidial morphogenesis, and DON biosynthesis in *F. graminearum in vitro*. The antifungal effects of guaiacol may be attributed to its capability to cause damage to the cell membrane by disrupting Ca^2+^ transport channels. Despite the observations in this study, many future works are still required. What is the relationship between Ca^2+^ transport and the growth and toxicity of *F. graminearum*? Does the antifungal activity of guaiacol against *F. graminearum* proceed through other mechanisms? Further understanding of these questions will accelerate the investigation of the fungicidal properties of guaiacol, which in turn will reveal the possible mechanisms and potential of this antioxidant in inhibiting mycotoxins in *F. graminearum*.

## Data Availability Statement

The original contributions presented in the study are included in the article/Supplementary Material, further inquiries can be directed to the corresponding author/s.

## Author Contributions

TG was involved in data acquisition, draft and critical revision of article, and final approval. YZ was involved in data analysis. JS and SM were involved in data acquisition and data consulting. JX and XL conceived and designed the manuscript. All authors read and approved the manuscript.

## Conflict of Interest

The authors declare that the research was conducted in the absence of any commercial or financial relationships that could be construed as a potential conflict of interest.

## Publisher’s Note

All claims expressed in this article are solely those of the authors and do not necessarily represent those of their affiliated organizations, or those of the publisher, the editors and the reviewers. Any product that may be evaluated in this article, or claim that may be made by its manufacturer, is not guaranteed or endorsed by the publisher.

## Footnotes

1
https://www.ncbi.nlm.nih.gov/
