# Supplementary material for: The Antioxidant Guaiacol Exerts Fungicidal Activity Against Fungal Growth and Deoxynivalenol Production in Fusarium graminearum
Source: Front Microbiol. 2021 Nov 15;12:762844. doi: 10.3389/fmicb.2021.762844 (PMC8634675; doi:10.3389/fmicb.2021.762844)
Supplement: Supplementary file 1 [file Table_1.doc]

**Supplementary material**

Additional Supporting Information may be found in the online version of this article:

**Table 1.** Oligonucleotide primers used in this study and their relevant characteristics.

| Primer^a^ | Sequence（5'-3'） | Use |
| --- | --- | --- |
| P1 | GTGTCGCCAACAGGAAAG | qRT-PCR primers for the *FGSG_00508* gene |
| P2 | GATCGCCAGTGGTGAAAA |
| P3 | CCAGTGCTGAAGCCAAAT | qRT-PCR primers for the *FGSG_04245* gene |
| P4 | AATAGCGGCGACGATAGA |
| P5 | CCAGTGCTGAAGCCAAAT | qRT-PCR primers for the *FGSG_04919* gene |
| P6 | CGACACCACCCTCAATCC |
| P7 | GCCACTGGTCTTTCTATG | qRT-PCR primers for the *FGSG_08985* gene |
| P8 | GTCGGAGCAAATGTTAGT |
| P9 | GAGTGTTTCATGCATGGCTACGTC | qRT-PCR primers for the *Tri5* gene |
| P10 | CTGAGCCTCCTTCACATCGTCC |
| P11 | TATCGAAAATTATATAACCACATC | qRT-PCR primers for the *Tri6* gene |
| P12 | CTGAGGGCATTCTGAGTAGCGACA |
| P13 | CTTACTGCCTCCACCAACTG | qRT-PCR primers for the *FGSG_08811* gene |
| P14 | TGACGTTGGAAGGAGCGAAG |
